# The Dry Season Shuffle: Gorges Provide Refugia for Animal Communities in Tropical Savannah Ecosystems

**DOI:** 10.1371/journal.pone.0131186

**Published:** 2015-07-02

**Authors:** J. Sean Doody, Simon Clulow, Geoff Kay, Domenic D’Amore, David Rhind, Steve Wilson, Ryan Ellis, Christina Castellano, Colin McHenry, Michelle Quayle, Kim Hands, Graeme Sawyer, Michael Bass

**Affiliations:** 1 Department of Ecology & Evolutionary Biology, University of Tennessee, Knoxville, Tennessee, United States of America; 2 School of Environmental and Life Sciences, University of Newcastle, Callaghan, New South Wales, Australia; 3 Fenner School of Environment and Society, Australian National University, Canberra, Australian Capital Territory, Australia; 4 Department of Natural Sciences, Daemen College, Amherst, New York, United States of America; 5 Department of Anatomy and Developmental Biology, Monash University, Clayton, Victoria, Australia; 6 Queensland Museum, South Brisbane, Queensland, Australia; 7 Department of Terrestrial Zoology, Western Australian Museum, Welshpool, Western Australia, Australia; 8 Utah’s Hogle Zoo, Salt Lake City, Utah, United States of America; 9 Stop the Toad Foundation, West Perth, Australia; 10 Frogwatch NT, Wagaman, Northern Territory, Australia; 11 El Questro Wilderness Park, Kununurra Western Australia, Australia; Estacion Experimental de Zonas Áridas (CSIC), SPAIN

## Abstract

In the wet-dry tropics, animal species face the major challenges of acquiring food, water or shelter during an extended dry season. Although large and conspicuous animals such as ungulates and waterfowl migrate to wetter areas during this time, little is known of how smaller and more cryptic animal species with less mobility meet these challenges. We fenced off the entire entrance of a gorge in the Australian tropical savanna, offering the unique opportunity to determine the composition and seasonal movement patterns of the small vertebrate community. The 1.7 km-long fence was converted to a trapline that was deployed for 18-21 days during the early dry season in each of two years, and paired traps on both sides of the fence allowed us to detect the direction of animal movements. We predicted that semi-aquatic species (e.g., frogs and turtles) would move upstream into the wetter gorge during the dry season, while more terrestrial species (e.g., lizards, snakes, mammals) would not. The trapline captured 1590 individual vertebrates comprising 60 species. There was a significant bias for captures on the outside of the fence compared to the inside for all species combined (outside/inside = 5.2, CI = 3.7-7.2), for all vertebrate classes, and for specific taxonomic groups. The opposite bias (inside/outside = 7.3, N= 25) for turtles during the early wet season suggested return migration heading into the wet season. Our study revealed that the small vertebrate community uses the gorge as a dry season refuge. The generality of this unreplicated finding could be tested by extending this type of survey to tropical savannahs worldwide. A better understanding of how small animals use the landscape is needed to reveal the size of buffer zones around wetlands required to protect both semi-aquatic and terrestrial fauna in gorges in tropical savannah woodland, and thus in ecosystems in general.

## Introduction

The wet-dry tropics are characterized by distinct wet and dry seasons and are found on every major continent. While the wet season is often plentiful, the dry season challenges animals to acquire resources or survive over many months with little to no precipitation. For many species, the wet-dry seasons drive an intra-year ‘boom to bust’ cycle which requires highly specialized ecological traits to evolve [1, 2]. Understanding how animals respond to dry season conditions is important—we would expect prolonged dry conditions to impact population dynamics and ultimately shape an organism’s physiology, ecology and behavior [3–5]. Revealing these responses is also critical for predicting the impacts of anthropogenic influences on species and population persistence. For example, climate change models predict increases in the length and intensity of dry seasons over large areas such as the Amazon Basin [6].

Generally, animals stressed by prolonged dry conditions can either migrate or aestivate during the dry season. Many species migrate with the seasonal rhythms in search of water and food, including African ungulates such as wildebeest, gazelles and zebras. As the dry season intensifies, they migrate hundreds of kilometers toward greener pastures, returning with the onset of the wet season several months later [7, 8]. Similarly, many waterfowl species utilize natural and artificial wetlands as alternative refugia as floodplains retract throughout the dry season [9–11].

Less well-known is how smaller and more cryptic vertebrates with less mobility meet the challenge of dry season conditions. Movements of five species of snakes followed retreating water levels during the dry season in seasonally-flooded wetlands [12, 13]. Not surprisingly, many semi-aquatic species that require rainfall and temporary wetlands to breed or feed will often aestivate underground (e.g., frogs, [14]. Fish communities and crocodilians use river channels and permanent lagoons as dry season refugia [15–17], and in smaller systems fish can escape drying conditions by seeking refuge in the burrows of other animals [18, 19]. Mosquitos use non-flowing water bodies as dry season refugia, from which they are able to migrate a few kilometers to feed [20].

Understanding dry season responses in smaller terrestrial vertebrates would be particularly invaluable in Australia, where terrestrial animal communities consist almost entirely of small to medium-sized vertebrates. For example, at a maximum size of <30 kg the dingo, *Canis lupus*, is Australia’s largest terrestrial predator [21]. The paucity of large native herbivores and predators in Australia focuses attention on small to medium-sized animals when considering the ecology and conservation of vertebrate communities (e.g., [22, 23].

We might expect heterogeneity in the responses of species to dry season conditions within a given animal community, given a diversity of life history strategies. For example, some species such as burrowing frogs might move less horizontally because they can burrow during dry conditions. A formal test of the dry season responses of an animal community would require studying movement patterns in a multitude of species spanning the early dry season (e.g., using VHF or GPS-based telemetry). Collectively such studies would be prohibitively costly, both financially and logistically. Alternatively, a large-scale pitfall trapline with paired traps on the inside and outside of the fence could detect any bias in movement direction of a wide range of species in an animal community. This method is often used in determining the timing of breeding in amphibians; a bias in capture rates on the outside of a fence indicates movement to the breeding site, whereas the opposite bias reveals post-breeding movements away from the breeding site [24].

We completely fenced off the entrance of a gorge in the Australian tropical savanna escarpment, as part of a conservation effort to exclude the invasive cane toad (*Rhinella marina*), offering a rare opportunity to extensively determine the composition of the small vertebrate community. The 1.7 km-long fence was converted into a pitfall trapline that was employed for 18–21 days during the early dry season in each of two years (2011 and 2012), and paired traps on both sides of the fence allowed us to detect the direction of animal movements. We hypothesized that aquatic species (e.g., non-burrowing frogs, turtles) would move upstream into the gorge during the dry season, while more terrestrial species (e.g., lizards, snakes, and mammals) would not. We discuss the potential for the generality of our findings for the Kimberley region, and for other similar systems in the wet-dry tropics.

## Methods

### Study area, fence conversion and trapping methods

Approvals for the research reported herein were obtained by the Department of Parks and Wildlife, Western Australia (SF009165) for animal research, and from the Animal Ethics Committee of the School of Environmental and Life Sciences, Newcastle University (A-2012-214). The site accessed is owned by El Questro Station and managed by Delaware North as the El Questro Wilderness Park; we received full approval to access the site. Threatened species were not captured or handled during the study.

The study was conducted at Emma Gorge, a 1.6 km-long sandstone gorge in the Cockburn Ranges of El Questro Wilderness Park, Western Australia (15°53’42.12” S, 128°7’56.84” E). The ecosystem is open savannah woodland and is situated in the wet-dry tropics. The dry season (May-October) receives very little rainfall (< 60 mm), compared to >800 mm of rain falling during the wet season (November-April) (Australian Bureau of Meteorology). The Cockburn ranges rise to ~400 m above the adjacent floodplain, and are dissected by numerous sandstone gorges.

**Fig 1:**
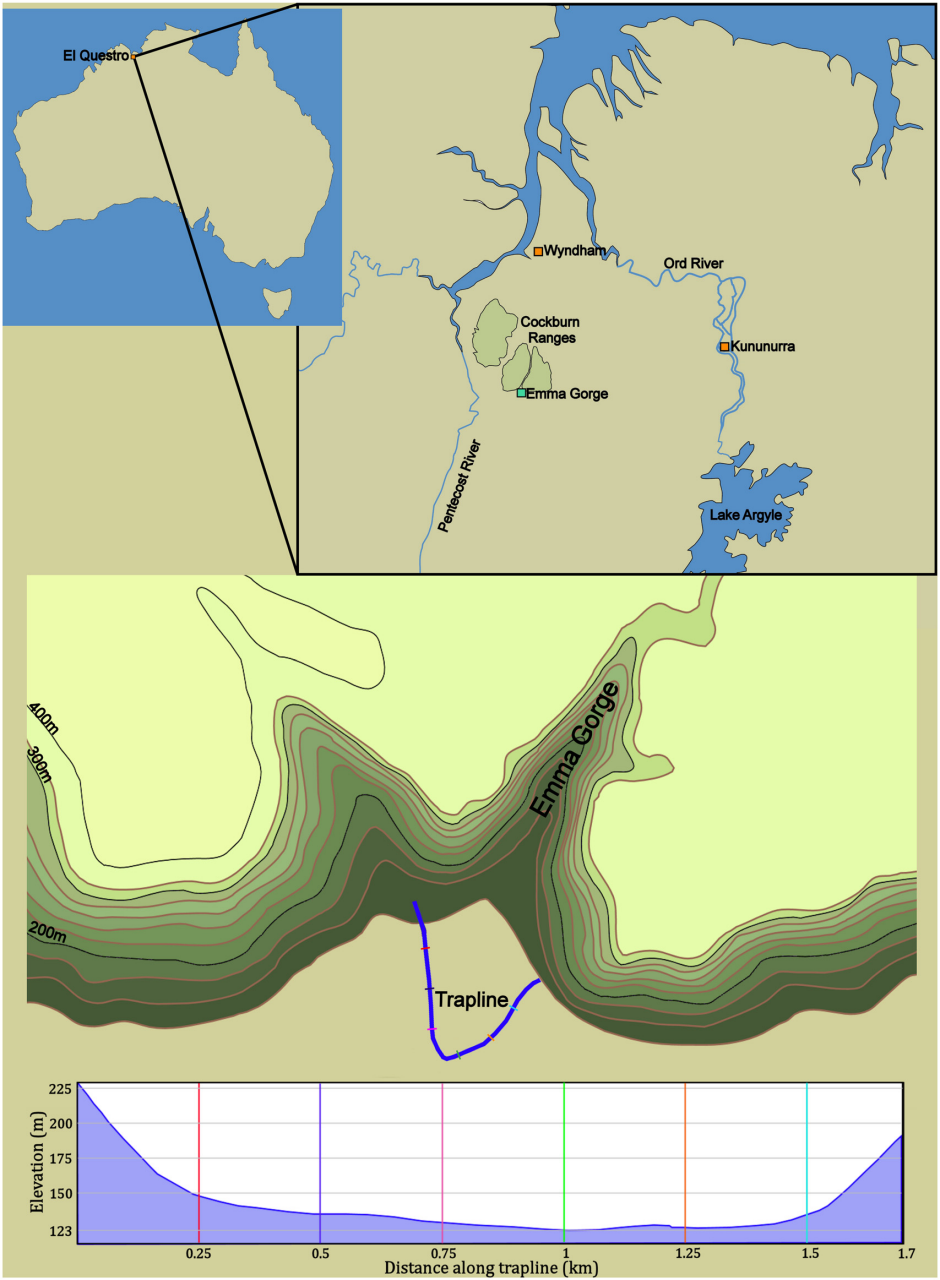
Study area, showing the 1.7 km-long pitfall trapline across the entrance to Emma Gorge, in Questro Wilderness Park, in northern Western Australia. Colors link points along the trapline with the elevation profile.

**Fig 2:**
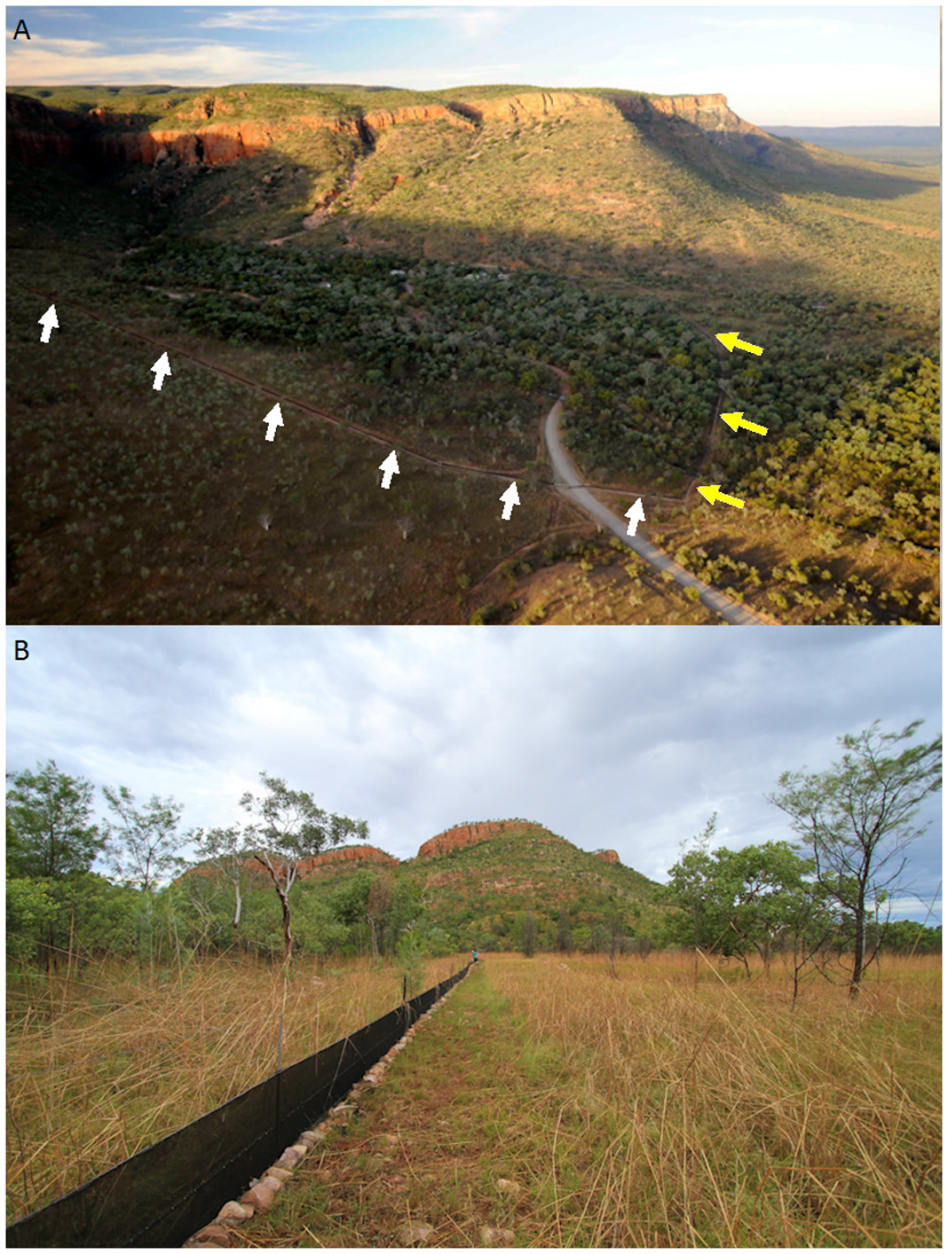
(A) Aerial view from the southwest of Emma Gorge, showing the 1.7 km-long trapline (arrows) crossing the dirt road into Emma Resort. Note that animals moving across the west wing (white arrows) of the trapline towards the gorge would be moving from drier savannah woodland (brownish) towards wetter riparian areas (greenish), while animals crossing the east wing (yellow arrows) towards the gorge would be moving from one riparian area to another. (B) Ground view of the west wing of the Emma Gorge trapline, showing the fence in savannah woodland in the foreground and the escarpment in the background.

The upstream part of Emma Gorge commences at a waterfall, whereas Emma Creek continues downstream between cliffs and scree slopes ranging ~100–200 m high. The creek averages < 0.5 m deep and splits into two channels that rejoin further downstream where water flows out of the gorge and into the low savannah woodland (Figs 1 and 2). During our study the open savannah grassland/woodland was dominated by a scattered overstory of cabbage gum (*Eucalyptus confertiflora*), northern salmon gum (*E*. *bigalerita*), Darwin woollybutt (*E*. *miniata*) and silky-leafed grevillea (*Grevillea pteridifolia*).

The entrance to Emma Gorge included a cattle fence that spanned 1.2 km across the alluvial floodplain, terminating at rocky hills associated with the gorge walls (Fig 2). In March 2011, volunteers for the Stop the Toad Foundation converted this fence into a cane toad (*Rhinella marina*) exclusion fence (Figs 1 and 2), using a design similar to that used to exlcude invasive cane toads from artificial waterways in the Northern Territory [25]. Conversion involved reinforcing the fence with 1 m-high shade cloth, attached to the fence using c-clips, and either buried in the ground or folded towards the outside and covered with rocks in situations where the substrate was too hard to penetrate with a shovel. In addition, the ends of the fence were extended up the gorge slopes by 0.3 km to the west and 0.2 km to the east, creating a 1.7 km-long fence. The ends of the fence terminated at cliffs (Fig 2). The fence crossed two branches of Emma Creek, a perennial system that remains running throughout the dry season.

In the present study the toad fence was converted into a trapline by adding 47 traps in 2011 (26 pitfall traps and 21 funnel traps) and 62 traps in 2012 (the same 26 pitfall traps and 36 funnel traps). Pitfalls consisted of buckets (20 liter, 31 cm dia X 41 cm high) sunk into the ground and funnel traps (75 cm L x 18 cm W x 18 cm H; W.A. Poultry Equipment, Baldivis, Western Australia) were placed against the fence. Traps were paired such that each trap on one side of the fence was mirrored by another of its type on the opposite side. Distance between traps varied from 10–30 m and was based on equal dispersion of available traps along the trapline. To prevent captured animals from desiccating and overheating, wet absorbent cloths were used in all traps and additional shade and protection was provided in buckets (plastic plates) and funnel traps (vegetation).

Traps were open for 24 hours and checked once a day between 0600 and 1000 hrs during the following periods: 2011: 18 days during 27 May-23 June; 2012: 21 days during 25–29 April, 3–8 May, and 30 May- 8 June. Captured animals were identified and immediately released on the opposite side of the fence from which they were captured, based on the assumption that animals were attempting to move to that side when they were captured. Animals were released in shade to prevent stress from desiccation.

Turtles (*Chelodina burrungandjii*) captured in the trapline were individually marked by filing notches in the carapacial edge as part of a long-term mark-recapture study, and were released at their point of capture. We hand captured turtles in the gorge (by snorkeling during the day and spotlighting at night) opportunistically 6–12 times each year from early April to late June. Captured turtles were individually marked as above and immediately released at their point of capture on the opposite side of the fence.

### Data Analyses and approvals

We analyzed the data for all species combined, and for taxonomic and behaviorally distinct groups to see how differently the groups behaved, and how widely applicable the general trend of moving into the gorge was. Three tiers of groups were investigated: tier 1 identified the different vertebrate classes (amphibians, reptiles and mammals); tier 2 groups indicated amphibian ecotype (obligate burrowers vs. ‘typical’ frog), mammal infraclass (eutherian vs marsupial), and reptile order/suborder (lizard, snake, turtle); tier 3 identified lizard family (Gekkonidae, Varanidae, Pygopodidae, Scincidae), and snake ecotype (aquatic vs terrestrial). Obligate burrowers included the ornate burrowing frog (*Platyplectrum ornatum*), the toadlet (*Uperoleia* spp.), and the northern spadefoot toad (*Notaden melanoscaphus*). These groups were separated on the rationale that obligate burrowers should be less inclined to seek refuge in wet gorges during the dry season (because they would be more inclined to burrow to escape the dry season). Metamorphic frogs were deleted from the analyses because they would be expected to be dispersing away from water, rather than in a random manner, under a null model (see S1 Table). Snakes were divided into semi-aquatic and terrestrial ecotypes using the rationale that semi-aquatic snakes (*Tropidonophis mairii*) are more dependent upon water, and so might be more inclined to seek refuge in wet gorges.

For each group, mean daily counts were calculated as the number of individuals of each species captured divided by the number of days the traps were open. To investigate differences in mean daily counts for each subgroup within a group between the inside and outside of the fence, a Generalised Linear Mixed Model (GLMM) with Poisson distribution and log link function was performed in SAS, with fixed effects for the full factorial model of trap position, year, and group to test if catch rates differed either side of the fence and whether they varied between years and/or between groups. Variation due to other sources of variation were not central to the research question so were modeled as random effects. The primary random term was associated with assessing the degree to which day to day variability differed between the two years. Three additional terms tested whether this day by year term varied by trap position, groups or the combination of trap position by group. If a random effect could be estimated it was kept in the model to ensure that fixed effects estimates would not be underestimated by ignoring associated random effects. Where models did not converge initially, random effects with the smallest level of variability were removed until the model converged. From the fitted model we obtained the mean daily counts along with ratios of counts from the outside divided by the inside of the fence for each of the animals groupings. The 95% confidence intervals of mean counts and the ratio of counts of outside/inside were determined from appropriate combinations of terms on the linear predictor (based on the log link function). The associated standard errors and confidence intervals were converted from the linear predictor to the original scale by exponentiation. As part of the modelling process, overdispersion was evaluated using the ratio of the Pearson Chi-Square/DF. Where the ratio exceeded one, we observed the effect of adding random intercept terms on the degree of overdispersion and if ratios returned to values close to ‘1’, we were satisfied that the random terms were sufficient to explain the overdispersion. Because we looked for multiple effects within 7 groups, we applied a Bonferroni correction whereby we set the significance level to α = 0.05/7 = 0.007 to control family wise error rate at α = 0.05. We determined the probability of a difference in mean number of daily captures between the inside and outside of the fence (P_i,o_) for a subgroup. Finally, we determined the probability of an interaction between subgroup type, and the difference in mean number of daily captures between the inside and outside of the fence (P_i,o*group_).

In the case of turtles only, where the number trapped on the inside of the fence was zero, the uncertainties for the mean counts (inside and outside) and the ratio (outside/inside) were determined by a Bayesian approach and expressed as 95% credible intervals. No p-value exists with the Bayesian approach; instead, Pr (0<8) = X means that the probability of ‘0’ being < ‘8’ = X. For the counts in or out, the credible intervals (c) were given using a conjugate uniform gamma prior Gamma (a = 1, b = 0) and the posterior of the Poisson parameter was given as Gamma (c+a, n+b), where the number of periods n was set to the number of trap days, 39. As the gammas for each sample follow a chi-squared distribution, the credible interval for the ratio was determined using an F distribution. The details for all these variables followed Lindley [26]. The central value for the ratio was estimated as the median using the F distribution. There were not enough captures for birds (n = 2) or dragon lizards (Agamidae, n = 2) to model these groups, and these data were therefore excluded from statistical analyses.

## Results

We captured 1590 individual vertebrates comprising 60 species, including amphibians (N = 1290, 13 spp.), reptiles (N = 274, 39 spp.) and mammals (N = 24, 7 spp.) (Table 1). We captured 719 individuals in 2011 and 871 individuals in 2012.

**Table 1:** Diversity of vertebrate species captured in the pitfall trapline at Emma Gorge during 2011–2012. The three tiers reflect groupings based on taxonomy or ecotype.

Species	Tier 1	Tier 2	Tier 3	2011	2012	Total
**AMPHIBIA Frogs**						
*Crinia bilingua*	amphibian	typical	-	131	325	456
*Limnodynastes lignarius*	amphibian	typical	-	0	6	6
*Litoria caerulea*	amphibian	typical	-	1	0	1
*Litoria nasuta*	amphibian	typical	-	82	73	155
*Litoria copelandi*	amphibian	typical	-	0	7	7
*Litoria inermis*	amphibian	typical	-	22	66	88
*Litoria pallida*	amphibian	typical	-	0	10	10
*Litoria rothi*	amphibian	typical	-	0	4	4
*Litoria rubella*	amphibian	typical	-	1	0	1
*Litoria wotjulumensis*	amphibian	typical	-	10	10	20
*Notaden melanoscaphus*	amphibian	burrowing	-	0	1	1
*Platyplectrum ornatum*	amphibian	burrowing	-	66	142	208
*Uperolia spp*.	amphibian	typical	-	263	70	333
**REPTILIA Lizards**						
*Diporiphora arnhemica*	reptile	lizard	dragon	1	0	1
*Ctenophorus caudicinctus*	reptile	lizard	dragon	1	0	1
*Crenadactylus ocellatus*	reptile	lizard	gecko	0	1	1
*Gehyra nana*	reptile	lizard	gecko	0	1	1
*Heteronotia binoei*	reptile	lizard	gecko	5	8	13
*Heteronotia planiceps*	reptile	lizard	gecko	2	0	2
*Varanus acanthurus*	reptile	lizard	goanna	1	1	2
*Varanus glebopalma*	reptile	lizard	goanna	2	1	3
*Varanus gouldi*	reptile	lizard	goanna	0	2	2
*Varanus scalaris*	reptile	lizard	goanna	3	8	11
*Varanus tristis*	reptile	lizard	goanna	1	0	1
*Delma tincta*	reptile	lizard	pygopod	2	0	2
*Delma borea*	reptile	lizard	pygopod	1	3	4
*Lialis burtonis*	reptile	lizard	pygopod	5	8	13
*Carlia amax*	reptile	lizard	skink	3	7	10
*Carlia gracilis*	reptile	lizard	skink	4	7	11
*Carlia rufilatus*	reptile	lizard	skink	5	0	5
*Carlia tricantha*	reptile	lizard	skink	2	6	8
*Cryptoblepharus metallicus*	reptile	lizard	skink	3	0	3
*Ctenotus halysis*	reptile	lizard	skink	0	1	1
*Ctenotus inornatus*	reptile	lizard	skink	24	14	38
*Ctenotus pantherinus*	reptile	lizard	skink	5	0	5
*Ctenotus robustus*	reptile	lizard	skink	13	9	22
*Ctenotus tantillus*	reptile	lizard	skink	1	0	0
*Eremiascincus isolepis*	reptile	lizard	skink	3	2	5
*Lerista borealis*	reptile	lizard	skink	2	0	2
*Menetia maini*	reptile	lizard	skink	8	3	11
*Morethia ruficauda*	reptile	lizard	skink	3	1	4
*Proablepharus tenuis*	reptile	lizard	skink	5	1	6
*Tiliqua scincoides*	reptile	lizard	skink	0	1	1
**Snakes**						
*Antaresia childreni*	reptile	snake	terrestrial	1	1	2
*Demansia papuensis*	reptile	snake	terrestrial	12	16	28
*Furina ornate*	reptile	snake	terrestrial	1	3	4
*Pseudechis australis*	reptile	snake	semi-aquatic	7	0	7
*Pseudechis weigli*	reptile	snake	terrestrial	1	8	9
*Ramphotyphlops guentheri*	reptile	snake	terrestrial	0	1	1
*Ramphotyphlops kimberleyensis*	reptile	snake	terrestrial	1	1	2
*Tropidonophis mairii*	reptile	snake	terrestrial	2	21	23
**Turtles**						
*Chelodina burrungandjii*	reptile	turtle	-	0	8	8
**Mammals**						
*Leggadina lakedownensis*	mammal	eutherian	-	3	2	5
*Mus musculus*	mammal	eutherian	-	0	6	6
*Planigale maculata*	mammal	marsupial	-	5	0	5
*Pseudomys delicatulus*	mammal	eutherian	-	1	0	1
*Pseudomys nanus*	mammal	eutherian	-	2	3	5
*Rattus tunneyi*	mammal	eutherian	-	0	1	1
*Zyzomys argurus*	mammal	eutherian	-	0	1	1
**Birds**						
*Coturnix ypsilophora*	bird	-	-	2	0	2

For all species combined, there was a significant bias for captures on the outside of the fence compared to captures on the inside of the fence (p < 0.001), with an overall ratio of outside/inside (O/I) of 5.2 (Table 2). For each of the tiered groups and subgroups, with the exception of marsupials, there was a bias towards captures on the outside of the fence, although in a number of cases the bias was not statistically significant (Table 2). There were no year effects for any groups/subgroups in any of the models.

**Table 2:** Mean daily captures on the inside and outside of the fence for all groups, and the ratios of outside/inside. All means are fitted model means [95% confidence intervals]. O/I = mean daily counts outside of fence/mean daily counts inside of fence, as defined by the fitted model means. P_i,o_ refers to the probability of a difference in mean number of daily captures between the inside and outside of the fence for a subgroup. P_i,o*group_ refers to the probability of an interaction between subgroup type, and the difference in mean number of daily captures between the inside and outside of the fence.

Group	Sub-group	Mean daily count inside [95% CI*]	Mean daily count outside [95% CI*]	O/I ratio	O/I [95% CI*]	P_i,o_	P_i,o*group_
All species		4.2 [2.7, 6.4]	21.6 [14.5, 32.2]	5.2	[3.7, 7.2]	<0.001	
vertebrate class	amphibian	2.5 [1.6, 3.7]	20.1 [14.1, 28.7]	8.1	[5.7, 11.6]	<0.001	
	mammal	0.4 [0.2, 0.9]	0.7 [0.3, 1.3]	1.8	[0.7, 4.5]	0.204	<0.001
	reptile	2.0 [1.3, 2.9]	4.4 [3.0, 6.3]	2.2	[1.5, 3.2]	<0.001	
amphibian ecotype	typical frog	1.6 [0.7, 2.0]	11.7 [7.3, 18.6]	10.1	[6.5, 15.6]	<0.001	0.093
	obligate burrower	1.1 [0.6, 2.0]	7.5 [4.7, 12.2]	6.7	[4.3, 10.5]	<0.001	
mammal infraorder	eutherian	0.5 [0.2, 1.1]	1.0 [0.6, 1.8]	2.2	[0.8, 5.9]	0.127	0.424
	marsupial	0.6 [0.2, 1.9]	0.6 [0.2, 1.9]	1.0	[0.2, 5.3]	0.974	
reptile order/suborder	lizard	1.9 [1.3, 2.6]	3.2 [2.4, 4.2]	1.7	[1.2, 2.5]	0.006	
	snake	0.5 [0.2, 0.8]	1.7 [1.2, 2.4]	3.7	[1.9, 7.1]	<0.001	0.033
	turtle	0.0 [0.0, 0.1]	0.2 [0.1, 0.4]	12.5^^^	[2.5,∞]	+	
lizard family	gekkonid	0.4 [0.1, 1.0]	0.6 [0.3, 1.3]	1.8	[0.5, 6.6]	0.343	
	varanid	0.4 [0.2, 1.0]	0.7 [0.4, 1.4]	1.7	[0.6, 4.8]	0.326	0.968
	pygopodid	0.3 [0.1, 1.0]	0.8 [0.5, 1.5]	2.5	[0.7, 8.6]	0.153	
	scincid	1.4 [1.0, 1.9]	2.8 [2.1, 3.6]	2.0	[1.3, 3.0]	0.001	
snake ecotype	semi-aquatic	0.3 [0.1, 0.9]	1.6 [1.0, 2.5]	4.8	[1.6, 14.2]	0.006	0.609
	terrestrial	0.4 [0.3, 0.8]	1.5 [1.1, 2.1]	3.4	[1.8, 6.6]	<0.001	

*Due to the alternative Bayesian approach used for turtles, 95% CI is expressed as 95% credible intervals for this group.

^+^The central value for the ratio for turtles was estimated as the median using the F distribution.

^^^No p-value exists due to the alternative Bayesian approach used (see Methods).

Amphibians were both trapped significantly more on the outside of the fence than the inside (p < 0.001), demonstrating a very strong bias (O/I = 8.1; Table 2). Both obligate burrowing frogs and ‘typical’ frogs showed a strong, significant bias for being captured on the outside of the fence (O/I = 6.7 and 10.1, respectively, p < 0.001 for both), and the bias for typical frogs was greater than that for obligate burrowing frogs, but not significantly so (p_i,o*group_ = 0.093; Table 2).

Reptiles showed the same pattern as amphibians, but to a lesser extent (p < 0.001; O/I = 2.2). Within reptiles, all orders/suborders showed significant biases towards the outside of the fence (Table 2; O/I = lizards 1.7, p = 0.006; snakes 3.7, p<0.001; turtles 12.5, probability that zero is less than 8 [Pr (0<8)] 8 = 0.999). There were, however, differences between the types, with the bias for turtles being much stronger (O/I = 12.5) than any other reptile type (and indeed, any other group or subgroup). When broken down further, only skinks out of all lizard subgroups were deemed to have a significant bias (O/I = 2.0; p = 0.001), although all lizard types showed some (non-significant) bias (Table 2; O/I = geckos, 1.8; goannas, 1.7; pygopods, 2.5). Skinks were considerably more numerous than the other lizard types. Both semi-aquatic and terrestrial snakes showed a significant and strong bias for being captured on the outside of the fence (O/I = 4.8, p < 0.001; 3.4, p = 0.006, respectively; Table 2), but the difference between the two was not significant (Table 2).

The bias for mammals (O/I = 1.8) was not statistically significant, possibly due to a modest sample size (N = 25, Table 2). When broken into mammal infraorder, the bias increased for eutherian mammals (O/I = 2.2) and was removed for marsupials (O/I = 1.0), although the bias for eutherian mammals remained non-significant due to low counts (p = 0.13; Table 2).

## Discussion

We demonstrated a strong bias in captures on the outside vs. the inside of an extensive trapline that cut-off an entire gorge entrance, revealing, for the first time, the mass migration of an entire community of small vertebrates into the gorge as the dry season progressed. This migration involved all classes of terrestrial vertebrate. The influx, combined with a larger bias in more aquatic and semi-aquatic species (e.g., turtles, ‘typical’ frogs and semi-aquatic snakes), implicate the dry season ‘retraction’ of water and moisture into the gorge as the cause of the mass movements. Moreover, additional observations indicated that the bias was reversed as the wet season began for turtles, indicating (return) movements out of the gorge during the wet season. Movements from one wet area to another wet area across part of the fence (i.e., along Emma Creek), combined with recaptures of turtles further within the gorge (Fig 1), shed doubt on the explanation that the bias simply reflected smaller-scale movements from small dry patches to small wet patches.

The interpretation of an influx of vertebrates into the gorge during the early dry season was strongly supported by the data. For each of the groups and subgroups, with the exception of marsupials, there was a bias towards captures on the outside of the fence (Table 2). Although the bias was not statistically significant for a few sub-groups, this was generally correlated with low samples sizes in those groups (see also confidence intervals in Table 2). Second, more aquatic or semi-aquatic species showed a stronger bias for being captured on the outside of the fence than less aquatic or semi-aquatic species, as demonstrated by higher O/I ratios in frogs vs. other vertebrates, turtles vs. other reptiles, typical frogs vs. obligate burrowing frogs, and semi-aquatic snakes vs. terrestrial snakes (Table 2). Third, there was no effect of year on our results; the strong bias for captures on the outside of the fence remained consistent across both years. This suggests that the mass migration was an annual event rather than a one-off phenomenon (attributable to a large wet season, for example). Finally, mark-recapture data for turtles indicate long distance movements up the gorge during the dry season, and subsequent movement out of the gorge during the late dry/early wet season. We recaptured four of the turtles (by hand) that were initially captured in the trapline, further upstream in the gorge. Three individuals were captured 1.5–1.6 km upstream of the fence on 7 May 2012, 7 May 2013, and 13 May 2013; another was captured 100 m upstream of the fence on 5 May 2012. Opportunistic captures of turtles by El Questro rangers (M. Bass, unpubl. data) during the onset of rainfall in the very late dry season and early wet season (October-December) in 2012 revealed a reverse bias, with 22 of 25 captures occurring on the inside of the fence (I/O = 7.3, N = 25). Most of these captures were near the two creek crossings. Our null model was no difference in numbers of animal captures between the inside and outside of the trapline. It could be argued, however, that we should see higher capture rates inside the fence due to a humidity gradient (wetter areas within the gorge). Regardless, we found higher capture rates on the outside of the fence. Collectively, our data suggest that the small vertebrate community could spend as much as 5–6 months in the gorge during the dry season, although further taxon-specific studies are needed to test this idea.

At first glance our data could be said to simply reflect short-distance movements (e.g., a few meters) from drier to wetter areas. Indeed, we do not know how far animals moved into the gorge, Figs 1 and 2). However, animals captured in the east wing of the fence, along Emma Creek, moved from one wet area to another (Fig 2). For example, in 2012 O/I ratios in the east wing (eastern half) of the trapline were 4.2 (N = 178) for all vertebrates, 4.6 (N = 134) for amphibians, 3.6 (N = 41) for reptiles, and 2.0 (N = 3) for mammals. Thus, our data support the idea that animals were moving further, following retracting water and ground moisture over a larger scale (see also Bernardino and Dalrymple 1992). However, movement data on individual species are needed to confirm how far individuals are moving. Also, our data are not sufficient to reveal the ultimate cause(s) for the movements—some species may move to wetter areas to prevent desiccation, while other may be tracking food resources. The influx of terrestrial species into the gorge was surprising and contrary to our initial hypothesis, and may be explained by species tracking their prey. For example, many of the snake species feed on frogs, and the former may be moving up the gorge to feed on the latter. Madsen and Shine [27] suggested that migrations of water pythons (*Liasis fuscus*) from a wetland to floodplain margins reflected similar movements of their chief prey species (*Rattus colletti*). Also surprising was the movement of obligate burrowing frogs across the landscape. These species do not generally feed or breed during the dry season, and aestivate underground during that time (it has been hypothesized that obligate burrowing frogs seek dry season refugia underground [28]). However, our data revealed that at least some individuals make horizontal movements into wetter areas prior to aestivation, raising questions about the simplicity of that hypothesis. Perhaps these frogs are seeking a particular soil texture or density to construct refuges within the gorge. Collectively, it appears that most species are moving into the gorge as a dry season refuge, but that their life histories, ecology and behavior may dictate how they use this refuge (i.e., to aestivate, become inactive, or continue to feed). Future studies documenting seasonal variation in movements with the different groups would allow a formal test of the refugia hypothesis.

**Fig 3:**
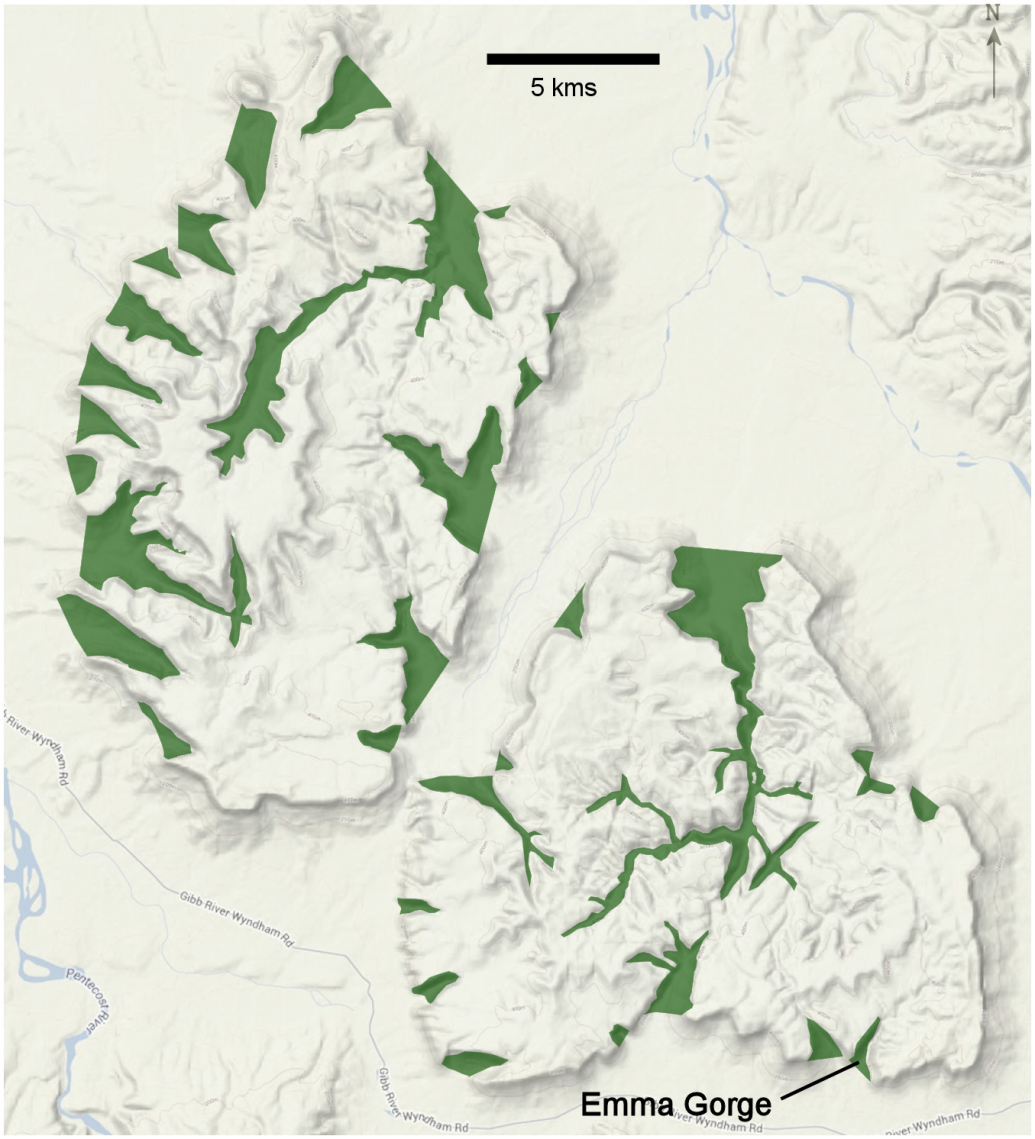
The Cockburn Ranges, showing Emma Gorge and ~40 other gorges (shaded) within the ~350 km^2^ area.

How general are our findings? There is little doubt that the mass movements we revealed reflect adaptations to endure the dry season, despite our inadequate knowledge of the extent of the movements and their ultimate function. Mortality of is often highest during the dry season (e.g., [5, 29]; thus, the dry season would be expected to influence population dynamics via natural selection. However, we only studied movements in one gorge. There are hundreds of sandstone gorges within the 423,000 km^2^ Kimberley region of northern Australia. In the ~350 km^2^ Cockburn Ranges alone there are > 40 gorges (Fig 3). Gorges across the Kimberley region likely provide refugia essential to the persistence of communities of small vertebrates in the surrounding tropical savannah woodland. It is likely that spring-fed creeks, rivers and other wetlands that persist during the dry season provide similar refugia for animals. However, many rocky gorges tend to retain water well into the early dry season due to high runoff and the gradual release of water from the surrounding rocky high ground. Gorge walls also provide cooler microhabitats via well-shaded areas and lush vegetation, including rainforest elements. Gorges may support animal communities with higher species diversity than communities using creeks and rivers as dry season refugia, a testable hypothesis within a comparative framework.

Considerable research and thought has been invested in understanding the size of buffer zones around wetlands needed to protect semi-aquatic species and assemblages using those wetlands [30, 31]. Our study extends that notion to terrestrial species, and reinforces the need for protecting the savannah woodland beyond the gorge entrances for semi-aquatic animals that reside within gorges (e.g., turtles). Currently, the Kimberley region is a wilderness area with very little land clearing or extensive habitat modification [32, 33]. Nevertheless, our study strongly suggests that an understanding of how animals use the landscape is needed to reveal the size of buffer zones around wetlands required to protect both semi-aquatic and terrestrial fauna in gorges in tropical savannah woodland, and thus in ecosystems in general.

## Supporting Information

S1 Table Raw capture data from the Emma Gorge fence trapline for 2011–2012. (XLSX)
